# Key Mechanisms in Lysosome Stability, Degradation and Repair

**DOI:** 10.1080/10985549.2025.2494762

**Published:** 2025-05-09

**Authors:** Rui Zhang, Marc A. Vooijs, Tom GH Keulers

**Affiliations:** Department of Radiation Oncology (MAASTRO)/GROW Research Institute for Oncology and Reproduction, Maastricht University Medical Center, Maastricht, The Netherlands

**Keywords:** Lysosome stabilization, ROS, lysosomal membrane permeabilization, lipid peroxidation, ESCRT, lysophagy

## Abstract

Lysosomes are organelles that play pivotal roles in macromolecule digestion, signal transduction, autophagy, and cellular homeostasis. Lysosome instability, including the inhibition of lysosomal intracellular activity and the leakage of their contents, is associated with various pathologies, including cancer, neurodegenerative diseases, inflammatory diseases and infections. These lysosomal-related pathologies highlight the significance of factors contributing to lysosomal dysfunction. The vulnerability of the lysosomal membrane and its components to internal and external stimuli make lysosomes particularly susceptible to damage. Cells are equipped with mechanisms to repair or degrade damaged lysosomes to prevent cell death. Understanding the factors influencing lysosome stabilization and damage repair is essential for developing effective therapeutic interventions for diseases. This review explores the factors affecting lysosome acidification, membrane integrity, and functional homeostasis and examines the underlying mechanisms of lysosomal damage repair. In addition, we summarize how various risk factors impact lysosomal activity and cell fate.

## Introduction

Lysosomes, discovered by Christian de Duve in the 1950s,^1^ are membrane-enclosed cytoplasmic organelles and serve as the main digestive compartments responsible for degrading biological macromolecules, including proteins, lipids, carbohydrates, and nucleic acids.^2^ Extracellular substrates targeted for degradation are delivered to lysosomes primarily through endocytic pathways, such as receptor-mediated endocytosis. In contrast, intracellular substrates, such as damaged mitochondria and protein aggregates, reach lysosomes mainly through autophagy, sequestered within autophagosomes that subsequently fuse with lysosomes to facilitate degradation. In addition to their degradative function, lysosomes have emerged as sophisticated signaling centers regulating cell growth, division, differentiation and cellular homeostasis.^3^

Lysosomal activity is driven by over 60 identified acid hydrolases, including proteases, nucleases, and lipases, which function optimally within the acidic environment of the lysosomal lumen (pH 4.5–5.5).^4^^,^^5^ These hydrolytic enzymes facilitate the breakdown of macromolecules, making lysosomes the cell’s primary degradation and recycling hub. The acidic pH is maintained by the vacuolar-type ATPase (V-ATPase), while integral membrane proteins such as lysosomal-associated membrane protein 1 (LAMP1) and lysosomal-associated membrane protein 2 (LAMP2) protect the lysosomal membrane from self-degradation.^6^

Dysfunctional lysosomes, caused by altered acidification, defective enzymes or compromised membrane integrity, are implicated in several diseases, including lysosomal storage diseases (LSD), including Gaucher disease, Niemann–Pick disease, and Tay–Sachs disease.

Lysosomal storage diseases (LSD) arise from enzyme deficiencies that hinder the breakdown of specific substrates within lysosomes, resulting in their accumulation and subsequent cellular and tissue dysfunction. For instance, cholesteryl ester storage disease (CESD) is an autosomal recessive lysosomal storage disorder caused by various mutations in the *lipase A* (*LIPA*) gene. These mutations lead to reduced activity of lysosomal acid lipase resulting in the accumulation of cholesteryl esters within lysosomes. When enzyme activity is minimal or absent, the condition manifests in infancy as Wolman disease, characterized by failure to thrive, malabsorption, hepatosplenomegaly, and rapid early mortality.^7^ Furthermore, destabilized lysosomal membranes and disruptions in autophagy contribute to autoimmune diseases, metabolic disorders, and kidney diseases.^8^ Understanding the main modulators of lysosome stability and damage repair mechanisms is crucial for gaining insights into lysosome-related diseases and developing novel therapeutic strategies.

## Biogenesis of Lysosomes

**Figure 1.**
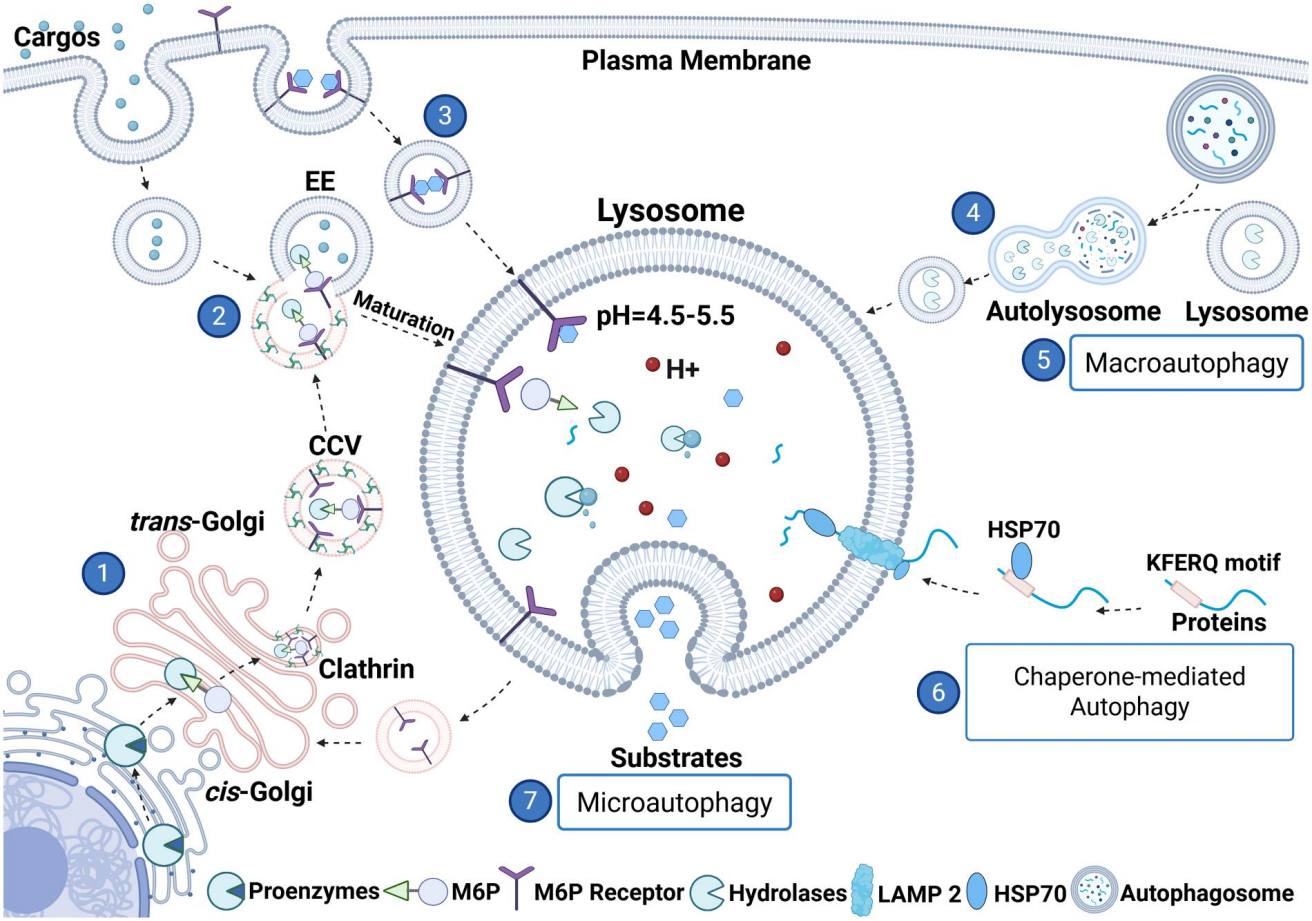
Lysosomal biogenesis and degradation pathways. (1) Lysosomal proenzymes are synthesized in the endoplasmic reticulum (ER) and modified in the *cis*-Golgi, where they are tagged with mannose-6-phosphate (M6P). In the *trans*-Golgi, M6P receptors (M6PRs) recognize M6P tagged proenzymes and mediate sorting into clathrin-coated vesicles (CCVs). (2) CCVs are transported to the early endosomes (EE), which act as the primary sorting hubs collecting internal and external cargo. As endosomes mature into lysosomes, the acidic environment triggers release of M6P-tagged hydrolases from their receptors and promotes their activation. (3) In addition, M6P receptors recognize extracellular or missorted M6P-tagged proteins, facilitating their internalization and transport to lysosomes through the endocytic pathway. (4) The remodeling and fission of autolysosomal membranes produces membrane fragments that mature into functional lysosomes. Lysosomes degrade cellular components sequestered through macroautophagy, microautophagy, and chaperone-mediated autophagy (CMA) or cargo that has been internalized through the endocytic pathway: (5) Macroautophagy delivers cargo through autophagosomes that fuse with lysosomes. (6) During chaperone mediated autophagy, HSP70 recognizes KFERQ-peptide containing proteins, which are unfolded and translocated to the lysosomal lumen through LAMP2.^125^ (7) Microautophagy involves direct lysosomal uptake via membrane invagination of the substrate. This image was created with BioRender.com.

Lysosomes arise from the endosomal system through a stepwise maturation process and rely on coordinated interactions with other intracellular organelles, which provide structural and functional components (Figure 1). Lysosome assembly begins with the synthesis of lysosomal membrane proteins and proenzymes in the endoplasmic reticulum (ER). These proteins are transported to the Golgi apparatus, where most of the enzymes are directly sorted into the endolysosomal system. Proenzymes destined for lysosomes pass through the *cis*-Golgi network, where they are tagged with mannose-6-phosphate groups (M6P).^9^ In the *trans*-Golgi network, M6P-tagged proenzymes bind to M6P receptors (MPR), after which they are sorted into clathrin-coated vesicles (CCV) for transport to the early endosomes (EE), which act as the primary sorting hubs collecting internal and external cargo. M6P-tagged proteins are trafficked into late endosomes (LE) through endosomal maturation. The acidic environment of lysosomes results in the dissociation of M6P from proenzymes, activating hydrolases. LE subsequently fuse with preexisting dense lysosomes, forming transient hybrid organelles known as endolysosomes (ELs). These ELs are active degradation sites and mature into classical dense lysosomes after substrate degradation.

Alternatively, proteins destined for lysosome biogenesis can be delivered indirectly to the lysosome via the plasma membrane. At the plasma membrane, M6P receptors are pivotal in recognizing secreted or mis-sorted M6P-tagged proteins, facilitating their internalization and transport to lysosomes through the endocytic pathway. In addition to proenzymes, lysosomal membrane proteins are targeted to lysosomes via specific sequence motifs. The clathrin-adaptor protein machinery recognizes short amino acid motifs (YXXΦ) located in the cytosolic regions of lysosomal membrane proteins. These motifs guide the transport of membrane proteins from the Golgi or plasma membrane to endosomes,^10^ ensuring proper lysosomal function.

In addition to *de novo* biogenesis, lysosomes can be generated through autophagic reformation. Through extensive membrane remodeling and fission, lysosomes are regenerated from autolysosomal membranes. This remodeling produces membrane fragments that mature into functional lysosomes, effectively replenishing the lysosomal pool and providing an efficient way to sustain lysosome availability, particularly during prolonged autophagic activity in response to nutrient deprivation (Figure 1).^11^^,^^12^

## Substrate Delivery to the Lysosome

Extracellular cargo destined for degradation, from either the extracellular environment or the cell surface, is delivered to the lysosome through the endocytic/phagocytic pathway (Figure 1). Endocytosis begins with the formation of small vesicles that bud inward from the plasma membrane and fuse with early endosomes (EE) upon internalization. These EE function as sorting hubs, directing cargo along distinct pathways. Cargo intended for recycling is routed back to the plasma membrane via recycling endosomes (RE). In contrast, cargo marked for degradation undergoes sorting and a series of maturation steps to form LE (pH 5.0–5.5).^13^ LE subsequently fuse with lysosomes to create EL, where degradation continues. Over time, ELs mature into classical dense lysosomes, completing the degradation process.

Autophagy is the central mechanism for the degradation and recycling of intracellular cargo , employing lysosomes to process larger cellular cargo such as protein aggregates, defective organelles such as mitochondria, and damaged lysosomes, facilitating their removal and supporting cellular homeostasis (Figure 1). During macroautophagy, cellular cargo is encapsulated by an autophagosome, which fuses with a lysosome, leading to the formation of autolysosomes.^14^ In contrast, chaperone-mediated autophagy directly targets single proteins and small protein aggregates to the lysosome via LAMP2 and heat shock protein 70 (HSP70), independent of the autophagosome.^15^ Recent studies demonstrate that autophagic substrates also can be directly internalized by lysosomes and LE through membrane protrusion and invagination, a process known as microautophagy ^16^ (Figure 1).

The lysosomal membrane is robust, in part due to the heavily glycosylated lysosomal integral membrane proteins (LIMPs) and lysosome-associated membrane proteins (LAMPs), which contribute to the formation of the glycocalyx, a protective lining that shields the membrane from luminal lytic enzymes. Yet, lysosomal membrane integrity can be compromised by a range of endogenous and exogenous factors, resulting in lysosomal membrane permeabilization (LMP) or complete membrane rupture. Cargo delivered to lysosomes, such as reactive oxygen species (ROS), aggregated proteins, crystalline substances, or indigestible particles, can impose physical or chemical stress. These factors can compromise lysosomal integrity, triggering the release of lysosomal contents into the cytoplasm and LMP-induced cell death including apoptosis.^17^ This can subsequently activate inflammatory pathways, impair cellular homeostasis and initiate cell death.^18^

This review provides a comprehensive analysis of the current literature on endogenous and exogenous factors that directly destabilize the lysosomal membrane, highlighting their molecular mechanisms, downstream effects, and potential implications for disease pathogenesis. In addition, lysosome stability and repair mechanisms are discussed.

## The Stability of Lysosomes

**Figure 2.**
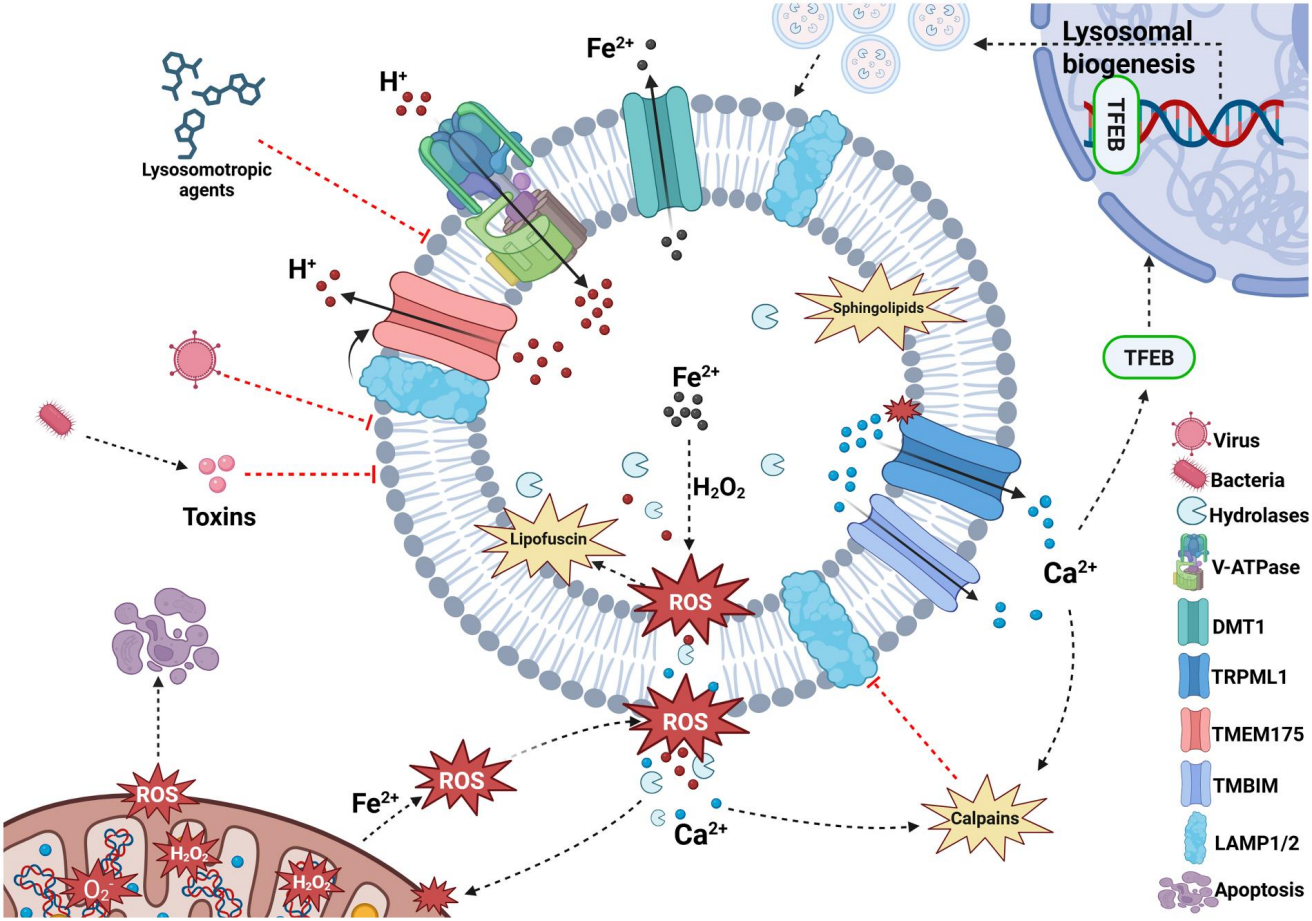
Factors affecting lysosome stability. Lysosomes are vulnerable to damage from reactive oxygen species (ROS). Defects in transporters, such as metal transporter DMT1, lead to iron accumulation and increased ROS production, resulting in lysosomal membrane peroxidation.^126^ This triggers the leakage of hydrolases, protons, and ions into the cytoplasm, disrupting mitochondrial oxidative respiration and further increasing ROS levels.^127^ In addition, ROS-induced damage leads to the accumulation of lipofuscin, which further contributes to membrane destabilization/disruption by promoting oxidative stress. The ROS sensor TRPML1 releases calcium into the cytoplasm upon activation, resulting lysosome biogenesis through TFEB signaling. Dysregulation of pH by proton influx and efflux pumps, such as V-ATPase and TMEM175 respectively, results in dysfunctional lysosomes. Increased calcium efflux through TRIMPL1 activates the calpain pathway, resulting in the degradation of lysosomal membrane proteins such as LAMP1 and LAMP2. In addition, lysosomotropic agents, such as chloroquine, ammonium chloride and antibiotics, accumulate in lysosomes and exhibit detergent-like properties, causing lysosomal membrane permeabilization (LMP). Proteins or metabolites from bacteria and viruses directly impair lysosomes by deglycosylating and degrading lysosomal membrane components. This image was created with BioRender.com.

Functional lysosomes require an intact membrane and a highly regulated internal environment, characterized by a low pH and a distinct ionic composition. When lysosomal membrane integrity is compromised, protons (H^+^) and other ions leak into the cytoplasm, disrupting intracellular pH homeostasis and ionic balance. Furthermore, abnormal changes in lysosomal components, such as iron overload, can alter membrane permeability and damage the lysosomal membrane. Below, we summarize the factors affecting lysosome stability from both intracellular and extracellular sources (Figure 2).

### ROS

Lysosomes are particularly prone to ROS-mediated damage due to their iron content, which facilitates the production of highly reactive hydroxyl radicals through Fenton-type reactions (H_2_O_2_+Fe^2+^→Fe^3+^+HO^•^ + OH^−^)^19^ (Figure 2). ROS are a group of unstable molecules, including hydrogen peroxide (H_2_O_2_), hydroxyl radical (OH^−^), singlet oxygen(^1^O_2_), and superoxide(O_2_^−^) that are produced by various cellular processes, of which the mitochondrial respiratory chain is the primary source of intracellular ROS.^20^^,^^21^ In mitochondria, superoxide dismutase (SOD) catalyzes the conversion of superoxide into H_2_O_2_, which can diffuse freely throughout the cell.^22^ Damaged mitochondria with a decreased membrane potential may result in a less efficient respiratory chain and enhanced ROS production. Without clearance, H_2_O_2_ can infiltrate lysosomes and participate in Fenton reactions.

Unlike mitochondria, lysosomes lack antioxidant enzymes such asSOD, catalase or glutathione peroxidase. Consequently, the accumulated ROS can readily permeate and damage the lysosomal membrane.^14^ Concurrently, leakage of ROS or cathepsins from the lysosome can in turn damage mitochondria and exacerbate cellular damage.^23^

ROS-induced damage to DNA, RNA, lipids, and proteins—through processes such as lipid peroxidation and amino acid oxidation—leads to the accumulation of lipofuscin, a nondegradable, membrane-bound waste product that accumulates in lysosomes ^24^ (Figure 2). Consequently, impaired autophagic turnover of dysfunctional mitochondria increases ROS levels, further promoting lipofuscin accumulation.^25^ In addition, lipofuscin accumulation enhances caspase-3 activity and promotes lysosomal membrane disruption, which is linked to the activation of the NLR family pyrin domain containing 3 (NLRP3) inflammasome and induction of necroptosis.^24^^,^^26^ It was reported that high levels of ROS can disturb lysosomal acidity and autophagic flux.^27^ Transient receptor potential mucolipin 1 (TRPML1) is an ion channel located on the membrane of lysosomes. It plays a crucial role as a ROS sensor within lysosomes. A slight increase in ROS can activate this channel, releasing lysosomal Ca^2+^ into the cytoplasm, which contributes to the activation of transcription factor EB (TFEB) signaling cascade,^28^ promoting lysosome biogenesis, autophagy and cellular adaptation to stress (Figure 2). In addition to indirect effects, ROS can directly damage the lipid bilayer of the lysosomal membrane, resulting in rupture.^29^^,^^30^ The release of lysosomal content into the cytoplasm might lead to apoptotic cell death due to the activation of caspases and other pro-apoptotic factors by lysosomal enzymes like cathepsins.^31^^,^^32^

### pH

Dysregulation of lysosomal pH has profound implications. pH deviations not only inhibit the hydrolytic activity of lysosomal enzymes but also disrupt lysosomal fusion, autophagic degradation, endocytic function, and transport. For instance, the loss of proteolytic activity impairs lysosome localization and motility, leading to autophagosome accumulation.^33^

Lysosome biogenesis relies on sorting lysosomal proteins from the *trans*-Golgi network to endosomes directly or indirectly via the plasma membrane. These endosomes undergo progressive maturation into LE and, eventually, lysosomes. The lysosomal pH, typically ranging from 4.5 to 5.0, is generated and maintained by V-ATPase^2^ and is essential for the optimal activity of lysosomal hydrolases, including proteases, nucleases, lipases, glucosidases, and other enzymes (Figure 2). Most proteinases are synthesized in the ER as inactive pro-enzymes containing a pro-peptide that is removed to activate the enzyme. This activation process requires an acidic environment.^34^

In addition to proton influx channels, transmembrane protein 175 (TMEM175) has been identified as a lysosomal proton efflux channel.^35^ Under physiological conditions, TMEM175 is activated by low pH (Figure 2). This protein helps maintain lysosomal pH homeostasis by counterbalancing the proton influx driven by V-ATPase. TMEM175 directly interacts with the lysosomal membrane proteins LAMP1 and LAMP2. This interaction inhibits proton efflux activity of TMEM175 and thereby contributes to maintaining an acidic pH in lysosomal organelles.^36^ Furthermore, other ion transporters may mediate proton flux and influence lysosomal pH. For example, solute carrier family 11 membrane 2 (SLC11A2/DMT1) is widely expressed, including in the lysosomal membrane, where it functions as an iron transporter coupled to a proton pump.^37^ In addition, lipid metabolism is highly pH-dependent. When lysosomal acid lipases are inhibited, they fail to degrade lipoprotein cholesteryl esters (CEs), causing an overload of CEs and increasing lysosomal luminal pH.^38^ Moreover, impaired lysosomal acidification triggers iron deficiency, which impairs both mitochondrial function^39^ and cell proliferation.^40^

### Ions

In addition to H^+^, the lysosomal lumen contains a variety of ions, including Na^+^, K^+^, Ca^2+^, Cl^−^ and Fe^2+^, each crucial for multiple physiological processes such as lysosomal condensation, lysosomal membrane potential, regulation of membrane trafficking, autophagy, membrane damage repair and secretion. Changes in ion concentrations can influence the structural and functional integrity of the lysosome. For instance, divalent metal transporter 1 (DMT1) is essential for exporting Fe^2+^ out of the lysosome and redistributing iron to vital cellular processes. Deletion of DMT1 results in the accumulation of Fe^2+^ within lysosomes, leading to lysosomal damage due to increased ROS formation and lipid peroxidation of the lysosomal membrane ^41^^,^^42^ (Figure 2).

In addition to the ER, lysosomes are also key calcium (Ca^2+^) storage organelles. Ca^2+^ signaling modulates several lysosomal processes, such as biogenesis, movement, lysosome repair, exocytosis, and fusion with other vesicles, and plays a crucial role in maintaining lysosomal membrane stability and function.^43–45^ Lysosomal-dependent cell death (LDCD) is a cell death pathway triggered by LMP. It is characterized by the release of lysosomal cathepsins and other hydrolases into the cytosol, which then triggers apoptosis and necroptosis.^46^ Ca^2+^ regulates several aspects of LDCD, including membrane permeabilization.

A key Ca^2+^-dependent pathway involved in LDCD is the Ca^2+^-calpain axis (Figure 2). Calpains are Ca^2+^-activated cysteine proteases that cleave structural lysosomal membrane proteins such as LAMP1 and LAMP2. This cleavage contributes to LMP^14^ and the release of lysosomal content into the cytosol. Massive lysosomal leakage increases cytosolic acidity, leads to uncontrolled digestion of cell components and cell death by necrosis. Partial and selective release of cathepsins, such as cathepsin B (CTSB) and -D (CTSD), activate a cascade of cell signaling events leading to cell death.^47^ Dysregulation of calpain activity and LDCD has been implicated in several pathologies, such as muscle atrophy and Alzheimer’s disease.^48^^,^^49^

Mucolipin TRP cation channel 1 (TRPML1) is the most studied calcium efflux channel in the lysosome. Through TRMPL1, calcium is released from the lysosome into the cytoplasm, which can be used for downstream processes. Activation of lysosomal TRPML1 increases the release of lysosomal Ca^2+^ into the cytosol, promoting the loss of mitochondrial membrane potential and apoptosis^43^ (Figure 2). Similarly, the cation channel transient receptor potential melastatin 2 (TRPM2) facilitates the release of lysosomal Ca^2+^ into the cytosol. Specifically, the activation of TRPM2 by ROS leads to LMP and the release of Zn^2+^ from lysosomes. The released Zn^2+^ is subsequently sequestered by mitochondria, triggering mitochondrial fission and resulting in fragmentation and dysfunction.^50^

In addition to TRP channels, members of the transmembrane BAX inhibitor-1 motif-containing (TMBIM) proteins play a role in calcium homeostasis. TMBIM family proteins only recognized not only as BAX inhibitors, but also as regulators of calcium signaling as well as regulators of lysosomal function.^51^ Among these, TMBIM1 functions as a pH-dependent calcium channel at the lysosomal membrane, modulating lysosomal acidification and calcium flux. Furthermore, TMBIM1 can cause LMP and mediate apoptosis by activating BAX in the mitochondria, a process also dependent on Ca^2+^.^52^ In summary, calcium can impair lysosomal integrity through multiple mechanisms. Ca^2+^ can impair lysosomal integrity directly by activating calpains, which results in the degradation of lysosomal membrane proteins, leading to LMP (Figure 2). Indirectly, dysregulated Ca^2+^ channels can cause cytosolic Ca^2+^ overload in the cytosol, ultimately triggering LDCD. Understanding these pathways provides valuable insights into lysosomal homeostasis and its impact on cell fate.

### Sphingolipids

Sphingolipids are a diverse family of lipids characterized by a sphingosine backbone. They serve as membrane lipids and play critical roles in signaling pathways, which modulate autophagy, apoptosis, and senescence, among other cellular processes.^53^ Major sphingolipids such as sphingomyelin (SM) and glycosphingolipids are key components of specific membrane microdomains that act as signaling platforms by interacting with specific membrane proteins. Sphingolipids are essential for lysosomal function, and disruptions in their composition can lead to lysosomal dysfunction. Studies show that sphingolipids tightly regulate lysosomal homeostasis and function, evidenced by sphingolipid-related diseases, including Gaucher disease and Niemann–Pick disease.^54^

Lysosomes rely on a delicate balance of sphingolipid metabolism and catabolism to sustain their function. This balance is achieved by sphingolipid synthesis and degradation, performed by lysosomal hydrolases, such as sphingomyelinase, which catalyzes the degradation of sphingomyelin into ceramide and phosphorylcholine, and acid β-glucosidase (GBA1), which hydrolyzes glucosylceramide into ceramide and glucose.^55^^,^^56^ Mutations or deficiencies in sphingolipid degradation pathways can result in the accumulation of sphingolipids, thereby compromising lysosomal stability and resulting in an array of pathologies, including a pro-inflammatory phenotype that promotes neurodegeneration. For instance, in Farber disease, a fatal lysosomal storage disorder, mutations in the ASAH1 gene encoding acid ceramidase lead to ceramide accumulation.^57^ Ceramide accumulation in lysosomes disrupts membrane stability by increasing permeability and causing membrane rupture ^58^ (Figure 2).

The structure of individual sphingolipid species influences the fluidity, structure and permeability of the membranes in which they reside.^59^^,^^60^ Failure to properly digest sphingolipids due to mutations in specific hydrolytic enzymes increases the risk of lysosomal damage.^61^^,^^62^ For instance, Niemann–Pick disease is characterized by sphingomyelin accumulation due to mutations in the acid sphingomyelinase gene. As a result, sphingomyelin accumulates inside lysosomes and permeabilizes the membrane in a detergent-like fashion.^63^

Furthermore, the accumulation of sphingolipids can lead to secondary defects such as protein aggregation and mitochondrial dysfunction.^64^ One way sphingolipids can inhibit lysosomal function is by disrupting the assembly of lysosomal membrane proteins. In yeast, for example, the disruption of sphingolipid synthesis leads to dissociation of a subdomain of V-ATPase,^65^ resulting in decreased V-ATPase activity and consequent lysosomal dysfunction.^66^ Given that V-ATPase function is conserved in mammalian lysosomes, similar disruptions could contribute to human diseases characterized by lysosomal dysfunction. In humans, defects in V-ATPase function are associated with disorders such as osteopetrosis, renal tubular acidosis and neurodegenerative diseases.^67^

In addition, sphingolipids affect lysosomal Ca^2+^ homeostasis. For instance, excess sphingosine, which accumulates in lysosomes due to sterol-binding protein deficiency, has been shown to impair lysosomal Ca^2+^ levels.^68^ This Ca^2+^ dysfunction arises from altered nicotinic acid adenine dinucleotide phosphate (NAADP)-a key pathway regulating lysosomal Ca^2+^ signaling. NAADP, a strong Ca^2+^-releasing second messenger, targets lysosomal Ca^2+^ channels to modulate Ca^2+^ levels required for proper endolysosomal trafficking.^69^ Furthermore, the accumulation of lysosomal sphingomyelin inhibits the activity of TRPML1,^70^ leading to impaired lysosomal trafficking.^71^

In addition to decreased degradation of sphingolipids, increased sphingolipid synthesis can contribute to lysosomal stress. For instance, the overactivation of synthases can increase sphingolipids.^72^ The undigested lipids form insoluble aggregates, leading to lysosomal expansion. Once the accumulation of sphingolipids exceeds the lysosome’s degradative capacity, lysosomal dysfunction may occur, including disrupted membrane integrity, impaired enzymatic activity or LMP, ultimately causing apoptosis.

### Lysosomotropic agents

Lysosomotropic agents, such as chloroquine, ammonium chloride, and antibiotics like quinolones, are weak bases that can easily cross cell membranes and accumulate within lysosomes. In the acidic environment, lysosomotropic agents become protonated, which traps them in the lysosomal lumen.^73^ High concentrations of protonated forms have detergent-like properties and can induce LMP and membrane rupture (Figure 2). Due to their specific effects on lysosomal functions and their phenotypic similarities to lysosomal storage disorders (LSDs), some lysosomotropic agents can be used to induce models of LSDs and to understand the mechanisms of lysosome-relevant diseases.^74^

The mechanisms by which lysosomotropic agents cause LMP vary depending on their chemical structures. For example, the accumulation of nonpermeable charged substances, such as free amino acids resulting from the metabolism of L-leucyl-L-leucine methyl ester (LLOMe), can generate osmotic pressure across the lysosomal membrane. This osmotic imbalance leads to water inflow, causing the lysosomes to swell and eventually rupture.^75^

Some lysosomotropic detergents can partition in the phospholipid bilayer and translocate across membranes as uncharged molecules. For instance, lysosomotropic amines, such as chloroquine and its derivatives, contain a moderately basic amino group that allows them to diffuse across cell membranes passively. After protonation, the amines embed their hydrophobic tail in the lysosomal membrane.^76^^,^^77^ Typically, this group of lysosomotropic drugs inhibits lysosomal lipid metabolism, autophagosomal degradation and sensitizes lysosomes to LMP.^78^

Although lysosomes are an integral part of the intracellular defense mechanism against pathogens such as bacteria, certain antibiotics can interfere with lysosomal function by accumulating within the lysosomal lumen, disrupting membrane integrity and cellular homeostasis. For instance, two quinolone antibiotics, norfloxacin and ciprofloxacin, are lipophilic bases accumulating in the lysosomal lumen and exert detergent-like effects on the lysosomal membrane.^79^ Similarly, gentamicin, an aminoglycoside antibiotic against bacterial infections, can induce acute nephrotoxicity in 5–25% of treated patients.^80^ Gentamicin is taken up by proximal tubular cells via adsorptive/receptor mediated endocytosis. As a result, gentamicin accumulates in the lysosomes, which induces local ROS production, resulting in LMP and, ultimately, apoptosis.^81^

In addition, several antipsychotic drugs (chlorpromazine, thioridazine, and aripiprazole) and antidepressants (desipramine, imipramine, and clomipramine) have been shown to exert cytotoxic effects by altering lysosomal membrane lipid composition or inducing lipid peroxidation through oxidative stress. These disruptions compromise lysosomal membrane integrity, making lysosomes more susceptible to LMP.^82^ In summary, many drugs, including antibiotics and psychotropic drugs, can cause severe side effects. Studies show that many drugs accumulate in the lysosomal lumen, disrupting membrane integrity. Understanding these off-target effects is essential for optimizing therapeutic strategies and mitigating unintended cellular toxicity.

### Bacteria and viruses

Many intracellular pathogens, including bacteria and viruses, enter host cells via phagocytosis and become enclosed within vacuoles, the endolysosomal system. To ensure their survival, these pathogens must escape from the endolysosomal system to avoid degradation within lysosomes. To establish infection and promote survival, they have evolved diverse strategies to escape lysosomal degradation, including destabilization of lysosomes through LMP or other mechanisms to compromise lysosomal integrity (Figure 2).

For instance, *Vibrio parahaemolyticus*, a member of the *Vibrio* genus, is a human pathogenic bacterium commonly found in saltwater environments. Its pathological effects stem from the secretion of VepA, a toxin that targets the V-ATPase complex in the lysosomal membrane, leading to lysosomal rupture in host cells. In a cell-free system, VepA was sufficient to induce the release of cathepsin D from isolated lysosomes, mediating lysosomal membrane permeabilization and subsequent cellular damage.^83^ *Streptococcus pneumoniae*, the most common cause of bacterial pneumonia, releases the toxin pneumolysin. In macrophages, pneumolysin contributes to LMP by the induction of pore formation. Interestingly, LMP activates apoptosis in macrophages rather than necrosis-like cell death ^84^ to limit bacterial spread. *Listeria* is a gram-positive pathogen responsible for human listeriosis. Its survival is facilitated by the secretion of the pore-forming toxin listeriolysin O (LLO). *Listeria* disrupts lysosomal integrity by increasing lysosomal pH, inducing LMP, and triggering the release of lysosomal contents, including cathepsins, in endothelial cells and macrophages.^85^^,^^86^ Some bacteria, including *Mycobacterium* produce sulfatides that modulate host immune responses and enhance their survival in macrophages, contributing to their virulence. The *Mycobacterium* specific sulfatide, sulfolipid SL-1, competes with 3-O-sulfogalactoceramide to bind to nucleotide oligomerization domain 2 (NOD2), thereby blocking autolysosome function through interaction with the V1B subunit of V-ATPase.^87^ In addition, cells infected with *Mycobacterium tuberculosis* produce 1-TbAd, which raises the vacuolar pH and inhibits acidic hydrolases, including lipases, inside the phagolysosome. Consequently, lipids, including cholesteryl esters and triglycerides, accumulate in the vacuole, mimicking lysosomal storage disease.^84^

In addition to bacteria, viruses also have sophisticated mechanisms to escape lysosomal degradation. Some viruses can impair the lysosome directly, or compromise lysosome stability through interaction with lysosome membrane components through mechanisms such as pore insertion, large-scale membrane disruption or lipid modification respectively.^88^ Although not damaging the lysosome directly, Hepatitis C virus escapes lysosomal degradation by targeting lectins in dendritic cells.^89^ Lectins are carbohydrate-binding proteins that play a crucial role in cellular and molecular recognition, including recognizing bacteria and viruses and targeting endolysosomal compartments such as lysosomes.

In contrast, the H5N1 influenza virus depends on the generation of neuraminidase. Neuraminidase can deglycosylate LAMP1, damaging the glycocalyx at the intraluminal side of the lysosome. By losing this protective layer, LAMP1 will be degraded by the intraluminal lysosomal hydrolase resulting in lysosomal membrane damage.^90^

The coronavirus SARS-CoV-2, responsible for coronavirus disease 2019 (COVID-19), was reported to develop unique adaptation, avoid lysosome-mediated destruction, and utilize lysosomes for viral replication. Coronavirus enters the cell mainly through clathrin-mediated endocytosis of the ACE-2 receptor. After internalization, acidification of compartments in the late-endosomal stage, potentially by fusion with lysosomes, liberates the virus and allows the viral genome’s release in the cytosol.^91^ Coronaviruses deacidify lysosomes to survive the highly hostile lysosomal environment by modulating H^+^ ion exchange across the lumen. Although the exact mechanism is unclear, a recent study suggests that the SARS-CoV-2 envelope protein acts as a proton channel, deacidifying late endosomal compartments, including lysosomes.^92^ This deacidification not only disrupts cellular processes like autophagy but also increases the risk of secondary bacterial infections, such as pneumonia, which is extremely common in patients with COVID-19.

In summary, bacteria and viruses are key factors interfering with lysosome stability through several mechanisms, such as pore formation and targeting the glycocalyx. Understanding these mechanisms provides valuable insights into host-pathogen interactions and may reveal potential therapeutic targets for combating infections and lysosomal dysfunction-related diseases.

### Others

Lysosomes have emerged as promising therapeutic targets in cancer treatment. Early clinical trials have demonstrated the feasibility and potential benefits of lysosomal inhibition across multiple cancer types.^93^^,^^94^ In addition to lysosomotropic agents such as (hydroxy)chloroquine, which are widely used to disrupt lysosomal function, photodynamic therapy (PDT) with photosensitizers offers a complementary approach to targeting lysosomes. Lysosomes play a central role in PDT-induced cell death, serving as key regulators of apoptotic signaling. Certain photosensitizers selectively accumulate in lysosomal membranes, leading to their destabilization upon activation and the subsequent release of proteolytic enzymes that amplify apoptotic cascades. For example, Pc13, a cationic zinc (II) phthalocyanine, induces cell photodamage through ROS generation, activating the mitochondrial apoptotic pathway by releasing lysosomal proteases.^95^ Similarly, the photosensitizer, NPe6, was reported to cause the immediate disruption of lysosomes, cathepsin-mediated cleavage of proapoptotic Bid, release of cytochrome c, and apoptosome activation after laser irradiation.^96^^,^^97^ ATX-s10, a structurally related photosensitizer, follows a comparable mechanism, initiating apoptosis through lysosomal damage.^98^ DNA-damaging drugs such as doxorubicin, carboplatin, and etoposide were found to cause LMP. This process is induced by p53-dependent BID upregulation and activation, followed by translocation of truncated BID to lysosomes.^99^

The P2X7 receptor, an ATP-gated ion channel, plays a crucial role in lysosomal stability and cell fate decisions. Upon activation, it mediates Ca^2+^ and Na^+^ influx and K^+^ efflux, which, in coordination with pannexin 1 channels, regulates calcium-dependent signaling pathways. One key downstream effect is the activation of TRPM2 channels on LE and lysosomes, triggering LMP and the release of cathepsins, leading to apoptotic or necrotic cell death.^100^

Furthermore, various chemical compounds and natural products have been shown to induce lysosomal dysfunction and are being explored for their potential to selectively eliminate cancer cells (reviewed in ^101^) For instance, silica nanoparticles accumulated in lysosomes, leading to inhibition of autophagy-mediated protein turnover.^102^ Various natural products, such as Pinus radiata bark extract, Omega 3 fatty acid docosahexaenoic acid, Monanchocidin A and others, possess anticancer properties, associated with LMP.^101^

## The Damage Repair of Lysosomes

**Figure 3.**
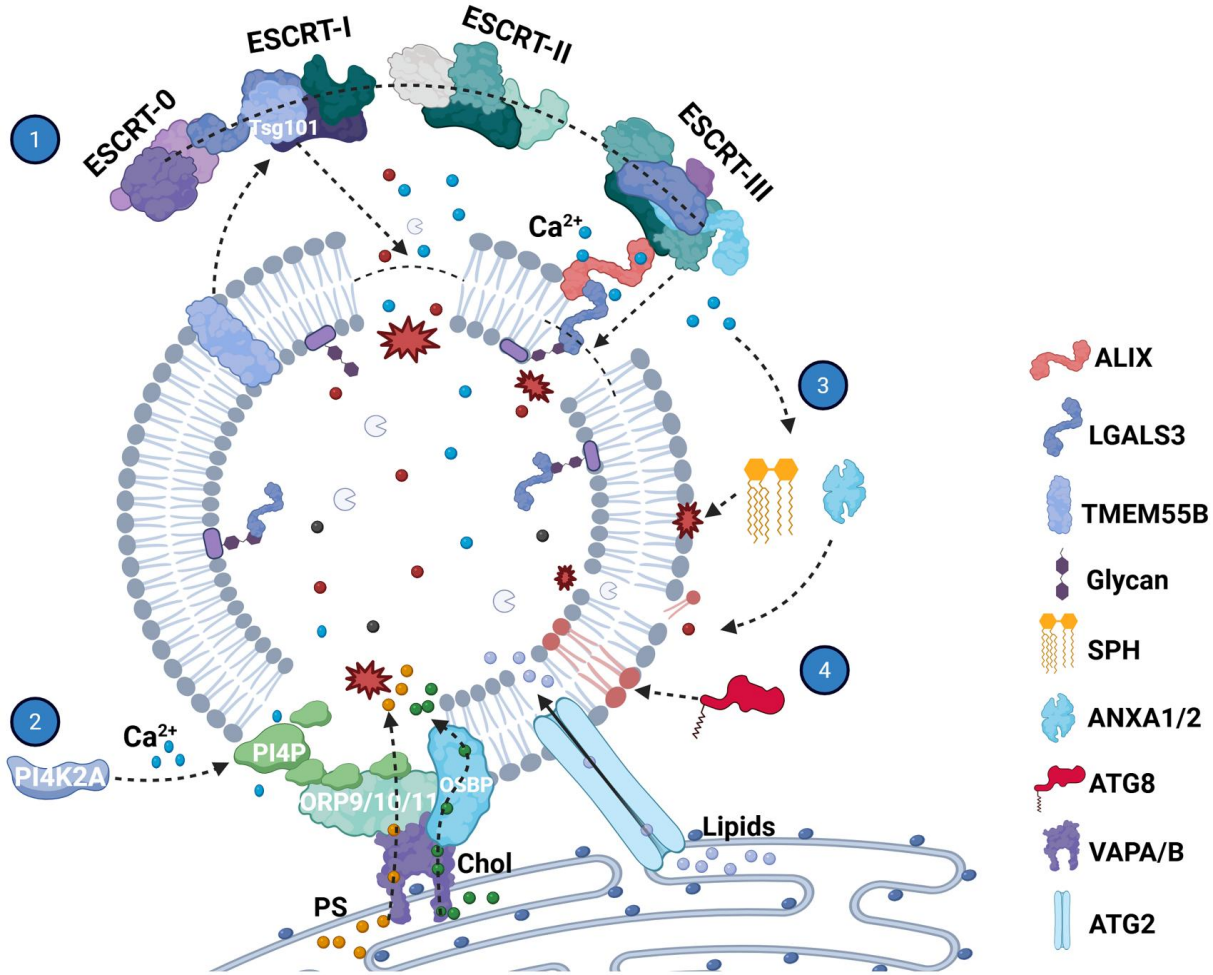
ESCRT-dependent and ESCRT-independent lysosome repair pathway. (1) Upon lysosome damage, ESCRT is recruited, dependent on Ca^2+^ and cofactors, such as Alix, galectin 3 and TMEM55B (PIP4P1). The ESCRT component TSG101 directly participates in lysosomal repair. (2) The phosphoinositide-initiated membrane tethering and lipid transport (PITT) pathway, without participant of ESCRT, is activated for rapid lysosomal repair. The Ca^2+^ release triggers the recruitment of phosphatidylinositol-4 kinase type2α (PI4K2A) to damaged lysosomes and generates high PI4P. Lysosomal PI4P subsequently recruits ORP9, ORP10 and ORP11, forming a bridge between the ER and lysosomal membrane. Phosphatidylserine (PS) is transported from ER to lysosome at ER-lysosome contact sites. Accumulation of PS activates the lipids transporter ATG2 to transport large amount of lipids into lysosome for direct repair. In addition, cholesterol (Chol) is transferred to the lysosome at ER contact sites through OSBP, assisting in membrane repair. (3) The lysosome damage also activates Ca^2+^-dependent repair pathways independent of ESCRT. Ca^2+^-activated scramblase catalyzes the scrambling and turnover of sphingolipids (SPH) to support repair. Annexins ANXA1 and ANXA2 are recruited by Ca^2+^ influx to minimize lysosomal leakage. (4) Atg8ylation is the process in which the LC3/GABARAP conjugation machinery is recruited to damage the membrane and mediate its remodeling. This image was created with BioRender.com.

Damaged lysosomes lose their ability to participate in metabolism, signaling, and cellular homeostasis, leading to disruptions in cellular function. This impairment can contribute to abnormal cell growth, proliferation, and differentiation, ultimately resulting in tissue damage and loss of cellular function. If damage is limited, cells can repair lysosomes by several mechanisms. However, damaged lysosomes beyond repair can be targeted for degradation. Several known mechanisms of lysosome repair will be discussed next (Figure 3).

The mechanism of lysosome repair involves both endosomal sorting complex required for transport (ESCRT)-dependent and ESCRT-independent pathways (Figure 3). ESCRT proteins, including complexes ESCRT-0,I,II and III, are key modulators of membrane remodeling during intraluminal vesicle biogenesis, cargo sorting and cytokinesis.^103^ The ESCRT-mediated repair occurs after minor damage to lysosomes. The efflux of Ca^2+^ from damaged lysosomes triggers the translocation of ESCRT components, activating the lipid-binding activity of the ESCRT component programmed cell death 6 interacting protein (PDCD6IP/Alix), which recruits ESCRT-III components.^104^ In addition, the recruitment of ESCRT can also rely on the ESCRT-I component tumor susceptibility 101 (TSG101) that lacks Ca^2+^binding activity.^105^ This membrane repair mechanism does not function independently and depends on co-factors’ involvement. For instance, LGALS3 recognizes damage-exposed glycans of the lysosome, required to efficiently recruit the ESCRT component Alix.^106^ The lysosomal protein phosphatidylinositol-4,5-bisphosphate 4-phosphatase 1 (PIP4P1) promotes the recruitment of components of the ESCRT machinery to lysosomal membranes to stimulate lysosomal repair.^107^

The ESCRT-independent pathway primarily relies on material recycling between the lysosome and the ER. One study identified a phosphoinositide-initiated membrane tethering and lipid transport (PITT) pathway without the participation of ESCRT.^108^ Upon LMP, phosphatidylinositol-4 kinase type2α (PI4K2A) rapidly accumulates on the damaged lysosomes, leading to the production of high levels of the lipid messenger phosphatidylinositol-4-phosphate (PI4P). Lysosomal phosphatidylinositol-4-phosphate, in turn, recruits various oxysterol-binding protein (OSBP)-related protein (ORP) family members, such as ORP9, ORP10, ORP11 and OSBP, to coordinate extensive new membrane contact sites between damaged lysosomes and the ER.^109^^,^^110^ The ORPs facilitate a robust transfer of phosphatidylserine and cholesterol from the ER to the lysosome, thereby promoting rapid repair of the lysosomal membrane.^111–114^ Ultimately, the lipid transfer protein autophagy-related 2 A (ATG 2 A), one of the mammalian autophagy core proteins, is recruited to damaged lysosomes and mediates rapid membrane repair through direct lysosomal lipid transfer.^115–117^ Another ESCRT-independent mechanism involves Ca^2+^-activated sphingomyelin scrambling and turnover, which clears minor lesions from the lysosome-limiting membrane and prevents cell death induced by lysosomal damage.^118^ In addition, the ubiquitously expressed annexins annexin 1(ANXA1) and annexin 2 (ANXA2) are recruited by Ca^2+^ influx to minimize leakage from damaged lysosomes.^119^

During lysosomal damage, another mammalian autophagy-related protein, MAP1LC3B, is rapidly and directly conjugated onto lysosome membranes. This process mediates lipid transfer and dynamics to repair lysosomes, representing a “non-canonical autophagy” pathway that helps maintain lysosomal integrity.^120^ Autophagy-related 5 (ATG5) is part of the E3 ligase directing the lipidation of LC3/GABARAP family proteins, a process central to membrane ATG8ylation and canonical autophagy. Loss of ATG5 increases the vulnerability of lysosomal membranes and inhibits repair machinery.^121^

## Lysophagy

**Figure 4.**
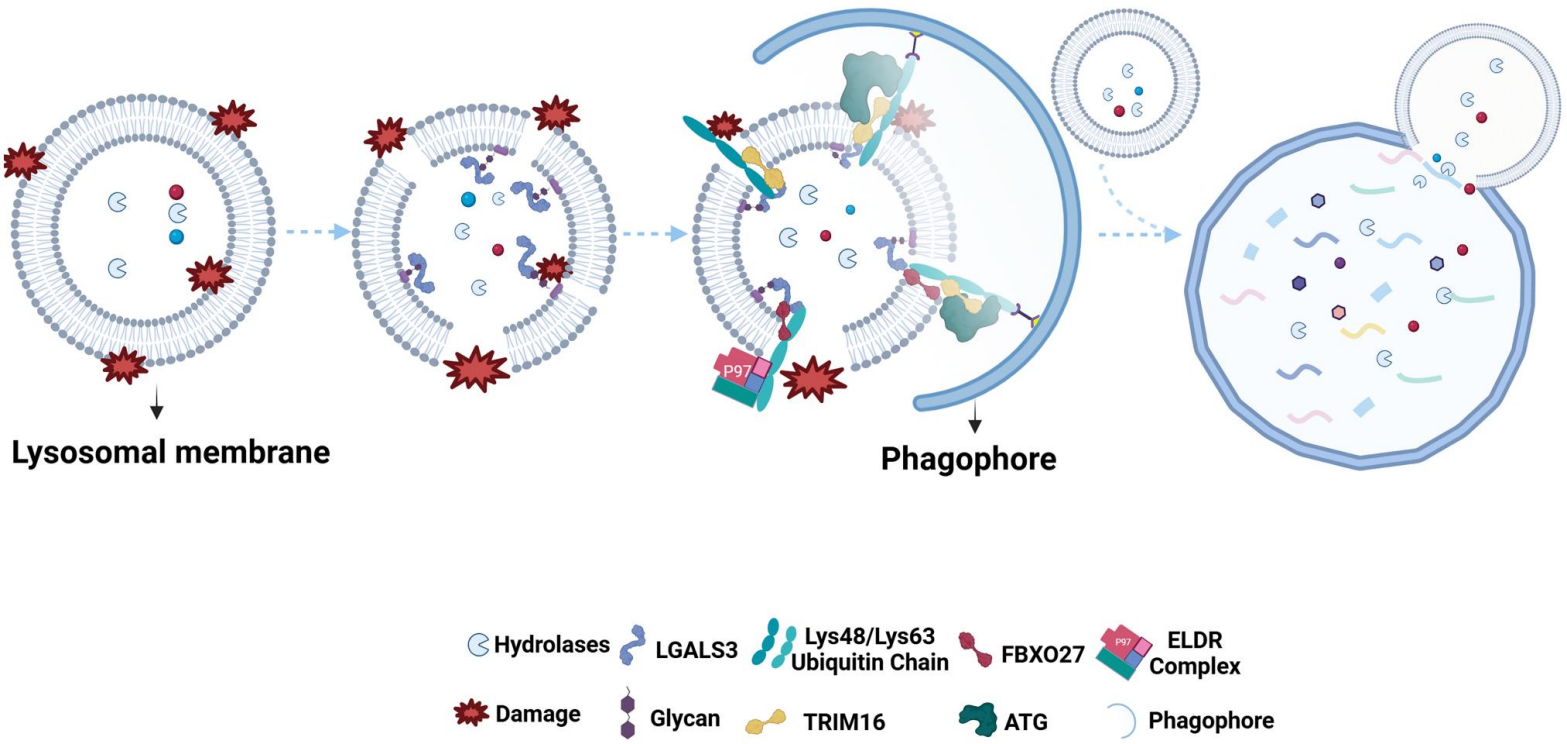
Lysophagy. Lysosomal membrane permeabilization leads to exposure of intraluminal glycans, which serve as the damage signals recognized by cytosolic LGALS3 and the ubiquitination machinery. The E3 ubiquitin ligases, TRIM16 and FBXO27 initiate the ubiquitination, followed by the formation of Lys63-linked and Lys48-linked ubiquitin chains. TRIM16 functions as a linker between damaged lysosomes and ATG proteins, allowing formation of the autophagosome membrane. Concurrently, the endolysosomal damage response (ELDR) complex is recruited to Lys48-ubiquitylated lysosomes, leading to Lys48-specific deubiquitylation and recruitment of LC3 to initiate autophagosome formation. Finally, fusion of autophagosomes with intact lysosomes results in the degradation of the damaged lysosomes. This image was created with BioRender.com.

Damaged lysosomes that cannot be repaired are targeted for degradation through a process known as lysophagy, which involves the turnover of lysosomes via autophagy. The general process of lysophagy includes the recognition of damaged lysosomes, ubiquitination of the lysosomal membrane, forming autophagosomes, and final degradation by intact lysosomes (Figure 4).

After lysosomal membrane damage, cytosolic LGALS3 enters the lysosomal lumen and binds to N-glycans. The E3 ubiquitin ligases tripartite motif containing 16 (TRIM16) and F-box protein 27 (FBXO27) then ubiquitylate the lysosomes.^122^^,^^123^ TRIM16 binds to LGALS3, acting as a bridge to recruit ATG proteins to damaged lysosomes, a key step in initiating lysophagy. Following ubiquitylation, the endolysosomal damage response (ELDR) complexes including deubiquitinating enzymes YOD1 and P97 deubiquitylate Lys48, promoting recruitment of LC3 to initiate lysophagy.^124^ Subsequently, autophagy receptors and additional regulators are recruited, leading to the formation of autophagosomes. Finally, damaged lysosomes are degraded by the fusion of autophagosomes with intact lysosomes. Within the whole process of lysophagy, the ubiquitylation/deubiquitylation dynamics occur on the lysosomal membrane and functions as the switch in the repair or degradation of damaged lysosomes.

## Concluding Remarks

Lysosomes are integral to cellular function and homeostasis and play critical roles in waste clearance, metabolism, and signaling. Their multifaceted nature connects them to various cellular processes and organelles, making them crucial and vulnerable. Damage to lysosomes can arise from various factors, including oxidative stress, pathogen interactions, or lysosome-damaging agents. This damage can lead to compromised lysosomal function and stability. Lysosomal damage impairs lysosomal activity and can trigger a cascade of adverse effects in other organelles, especially mitochondria.

Understanding the mechanisms of lysosomal damage and repair is essential for developing strategies to prevent and treat diseases associated with lysosomal dysfunction. In addition, insights into lysosomal dysfunction can lead to new therapeutic approaches by identifying novel targets and improving treatment strategies. This deeper understanding can enhance the precision and effectiveness of tailored therapies, offering new actionable interventions to address lysosomal diseases.

## Data Availability

The data that support the findings of this study are openly available in Science Data Bank at https://doi.org/10.57760/sciencedb.17206.

## References

[1] De Duve C, Pressman BC, Gianetto R, Wattiaux R, Appelmans F. Tissue fractionation studies. 6. Intracellular distribution patterns of enzymes in rat-liver tissue. Biochem J. 1955;60:604–617. PMID: 13249955. doi:10.1042/bj0600604. 13249955 PMC1216159

[2] Trivedi PC, Bartlett JJ, Pulinilkunnil T. Lysosomal biology and function: modern view of cellular debris bin. Cells. 2020;9:1131. PMID: 32375321. doi:10.3390/cells9051131. 32375321 PMC7290337

[3] Patra S, Patil S, Klionsky DJ, Bhutia SK. Lysosome signaling in cell survival and programmed cell death for cellular homeostasis. J Cell Physiol. 2023;238:287–305. PMID: 36502521. doi:10.1002/jcp.30928. 36502521

[4] Repnik U, Stoka V, Turk V, Turk B. Lysosomes and lysosomal cathepsins in cell death. Biochim Biophys Acta. 2012;1824:22–33. doi:10.1016/j.bbapap.2011.08.016. 21914490

[5] Bright NA, Davis LJ, Luzio JP. Endolysosomes are the principal intracellular sites of acid hydrolase activity. Curr Biol. 2016;26:2233–2245. PMID: 27498570. doi:10.1016/j.cub.2016.06.046. 27498570 PMC5026700

[6] Mindell JA. Lysosomal acidification mechanisms. Annu Rev Physiol. 2012;74:69–86. PMID: 22335796. doi:10.1146/annurev-physiol-012110-142317. 22335796

[7] Reynolds T. Cholesteryl ester storage disease: A rare and possibly treatable cause of premature vascular disease and cirrhosis. J Clin Pathol. 2013;66:918–923. PMID: 23999269. doi:10.1136/jclinpath-2012-201302. 23999269

[8] Gros F, Muller S. The role of lysosomes in metabolic and autoimmune diseases. Nat Rev Nephrol. 2023;19:366–383. doi:10.1038/s41581-023-00692-2. 36894628

[9] Braulke T, Bonifacino JS. Sorting of lysosomal proteins. Biochim Biophys Acta. 2009;1793:605–614. doi:10.1016/j.bbamcr.2008.10.016. 19046998

[10] Robinson MS. Adaptable adaptors for coated vesicles. Trends Cell Biol. 2004;14:167–174. doi: 10.1016/j.tcb.2004.02.002. 15066634

[11] Rodgers SJ, Jones EI, Arumugam S, Hamila SA, Danne J, Gurung R, Eramo MJ, Nanayakkara R, Ramm G, McGrath MJ, et al. Endosome maturation links PI3Kα signaling to lysosome repopulation during basal autophagy. EMBO J. 2022;41:e110398. doi:10.15252/embj.2021110398. 35968799 PMC9531306

[12] Dubnikov T, Ben-Gedalya T, Cohen E. Protein quality control in health and disease. Cold Spring Harb Perspect Biol. 2017;9:a023523. PMID: 27864315. doi:10.1101/cshperspect.a023523. 27864315 PMC5334259

[13] Scott CC, Gruenberg J. Ion flux and the function of endosomes and lysosomes: Ph is just the start: The flux of ions across endosomal membranes influences endosome function not only through regulation of the luminal ph. Bioessays. 2011;33:103–110. PMID: 21140470. doi:10.1002/bies.201000108. 21140470

[14] Wang F, Gómez-Sintes R, Boya P. Lysosomal membrane permeabilization and cell death. Traffic. 2018;19:918–931. PMID: 30125440. doi:10.1111/tra.12613. 30125440

[15] Cuervo AM, Dice JF. A receptor for the selective uptake and degradation of proteins by lysosomes. Science. 1996;273:501–503. PMID: 8662539. doi:10.1126/science.273.5274.501. 8662539

[16] Wang L, Klionsky DJ, Shen H-M. The emerging mechanisms and functions of microautophagy. Nat Rev Mol Cell Biol. 2023;24:186–203. doi:10.1038/s41580-022-00529-z. 36097284

[17] Eriksson I, Wäster P, Öllinger K. Restoration of lysosomal function after damage is accompanied by recycling of lysosomal membrane proteins. Cell Death Dis. 2020;11:370. PMID: 32409651. doi:10.1038/s41419-020-2527-8. 32409651 PMC7224388

[18] Boya P, Kroemer G. Lysosomal membrane permeabilization in cell death. Oncogene. 2008;27:6434–6451. PMID: 18955971. doi:10.1038/onc.2008.310. 18955971

[19] Chen Y, Yang Z, Wang S, Ma Q, Li L, Wu X, Guo Q, Tao L, Shen X. Boosting ros-mediated lysosomal membrane permeabilization for cancer ferroptosis therapy. Adv Healthc Mater. 2023;12:e2202150. PMID: 36408929. doi:10.1002/adhm.202202150. 36408929

[20] Bedard K, Krause KH. The nox family of ros-generating nadph oxidases: physiology and pathophysiology. Physiol Rev. 2007;87:245–313. PMID: 17237347. doi:10.1152/physrev.00044.2005. 17237347

[21] Yang S, Lian G. Ros and diseases: role in metabolism and energy supply. Mol Cell Biochem. 2020;467:1–12. PMID: 31813106. doi:10.1007/s11010-019-03667-9. 31813106 PMC7089381

[22] Terman A, Kurz T, Navratil M, Arriaga EA, Brunk UT. Mitochondrial turnover and aging of long-lived postmitotic cells: the mitochondrial-lysosomal axis theory of aging. Antioxid Redox Signal. 2010;12:503–535. PMID: 19650712. doi:10.1089/ars.2009.2598. 19650712 PMC2861545

[23] Papadopoulos C, Meyer H. Detection and clearance of damaged lysosomes by the endo-lysosomal damage response and lysophagy. Curr Biol. 2017;27:R1330–r1341. PMID: 29257971. doi:10.1016/j.cub.2017.11.012. 29257971

[24] Höhn A, Jung T, Grimm S, Grune T. Lipofuscin-bound iron is a major intracellular source of oxidants: role in senescent cells. Free Radic Biol Med. 2010;48:1100–1108. PMID: 20116426. doi:10.1016/j.freeradbiomed.2010.01.030. 20116426

[25] Moreno-García A, Kun A, Calero O, Medina M, Calero M. An overview of the role of lipofuscin in age-related neurodegeneration. Front Neurosci. 2018;12:464. PMID: 30026686. doi:10.3389/fnins.2018.00464. 30026686 PMC6041410

[26] Pan C, Banerjee K, Lehmann GL, Almeida D, Hajjar KA, Benedicto I, Jiang Z, Radu RA, Thompson DH, Rodriguez-Boulan E, et al. Lipofuscin causes atypical necroptosis through lysosomal membrane permeabilization. Proc Natl Acad Sci U S A. 2021;118:e2100122118. PMID: 34782457. doi:10.1073/pnas.2100122118. PMC8617501 34782457

[27] Song SB, Hwang ES. High levels of ros impair lysosomal acidity and autophagy flux in glucose-deprived fibroblasts by activating atm and erk pathways. Biomolecules. 2020;10:761. PMID: 32414146. doi:10.3390/biom10050761. 32414146 PMC7277562

[28] Zhang X, Cheng X, Yu L, Yang J, Calvo R, Patnaik S, Hu X, Gao Q, Yang M, Lawas M, et al. Mcoln1 is a ros sensor in lysosomes that regulates autophagy. Nat Commun. 2016;7:12109. PMID: 27357649. doi:10.1038/ncomms12109. 27357649 PMC4931332

[29] Saffi GT, Tang E, Mamand S, Inpanathan S, Fountain A, Salmena L, Botelho RJ. Reactive oxygen species prevent lysosome coalescence during pikfyve inhibition. PLoS One. 2021;16:e0259313. PMID: 34813622. doi:10.1371/journal.pone.0259313. 34813622 PMC8610251

[30] Sies H, Belousov VV, Chandel NS, Davies MJ, Jones DP, Mann GE, Murphy MP, Yamamoto M, Winterbourn C. Defining roles of specific reactive oxygen species (ros) in cell biology and physiology. Nat Rev Mol Cell Biol. 2022;23:499–515. PMID: 35190722. doi:10.1038/s41580-022-00456-z. 35190722

[31] Zhu SY, Yao RQ, Li YX, Zhao PY, Ren C, Du XH, Yao YM. Lysosomal quality control of cell fate: A novel therapeutic target for human diseases. Cell Death Dis. 2020;11:817. PMID: 32999282. doi:10.1038/s41419-020-03032-5. 32999282 PMC7528093

[32] Butler D, Bahr BA. Oxidative stress and lysosomes: CNS-related consequences and implications for lysosomal enhancement strategies and induction of autophagy. Antioxid Redox Signal. 2006;8:185–196. PMID: 16487052. doi:10.1089/ars.2006.8.185. 16487052

[33] Yogalingam G, Pendergast AM. Abl kinases regulate autophagy by promoting the trafficking and function of lysosomal components. J Biol Chem. 2008;283:35941–35953. PMID: 18945674. doi:10.1074/jbc.M804543200. 18945674 PMC2602914

[34] Richo GR, Conner GE. Structural requirements of procathepsin D activation and maturation. J Biol Chem. 1994;269:14806–14812. PMID: 8182087. doi:10.1016/S0021-9258(17)36696-6. 8182087

[35] Ratto E, Chowdhury SR, Siefert NS, Schneider M, Wittmann M, Helm D, Palm W. Direct control of lysosomal catabolic activity by MTORC1 through regulation of V-ATPase assembly. Nat Commun. 2022;13:4848. PMID: 35977928. doi:10.1038/s41467-022-32515-6. 35977928 PMC9385660

[36] Zhang J, Zeng W, Han Y, Lee WR, Liou J, Jiang Y. Lysosomal LAMP proteins regulate lysosomal pH by direct inhibition of the TMEM175 channel. Mol Cell. 2023;83:2524–2539.e7. e2527 PMID: 37390818. doi:10.1016/j.molcel.2023.06.004. 37390818 PMC10528928

[37] Shawki A, Knight PB, Maliken BD, Niespodzany EJ, Mackenzie B. H(+)-coupled divalent metal-ion transporter-1: functional properties, physiological roles and therapeutics. Curr Top Membr. 2012;70:169–214. PMID: 23177986. doi:10.1016/b978-0-12-394316-3.00005-3. 23177986 PMC7027397

[38] Dubland JA, Francis GA. Lysosomal acid lipase: at the crossroads of normal and atherogenic cholesterol metabolism. Front Cell Dev Biol. 2015;3:3. PMID: 25699256. doi:10.3389/fcell.2015.00003. 25699256 PMC4313778

[39] Yambire KF, Rostosky C, Watanabe T, Pacheu-Grau D, Torres-Odio S, Sanchez-Guerrero A, Senderovich O, Meyron-Holtz EG, Milosevic I, Frahm J, et al. Impaired lysosomal acidification triggers iron deficiency and inflammation in vivo. Elife. 2019;8:e51031. PMID: 31793879. doi:10.7554/eLife.51031. PMC6917501 31793879

[40] Weber RA, Yen FS, Nicholson SPV, Alwaseem H, Bayraktar EC, Alam M, Timson RC, La K, Abu-Remaileh M, Molina H, et al. Maintaining iron homeostasis is the key role of lysosomal acidity for cell proliferation. Mol Cell. 2020;77:645–655.e7. PMID: 31983508. doi:10.1016/j.molcel.2020.01.003. 31983508 PMC7176020

[41] Dixon SJ, Lemberg KM, Lamprecht MR, Skouta R, Zaitsev EM, Gleason CE, Patel DN, Bauer AJ, Cantley AM, Yang WS, et al. Ferroptosis: an iron-dependent form of nonapoptotic cell death. Cell. 2012;149:1060–1072. PMID: 22632970. doi:10.1016/j.cell.2012.03.042. 22632970 PMC3367386

[42] Hounjet J, Groot AJ, Piepers JP, Kranenburg O, Zwijnenburg DA, Rapino FA, Koster JB, Kampen KR, Vooijs MA. Iron-responsive element of divalent metal transporter 1 (DMT1) controls notch-mediated cell fates. FEBS J. 2023;290:5811–5834. PMID: 37646174. doi:10.1111/febs.16946. 37646174

[43] Siow WX, Kabiri Y, Tang R, Chao YK, Plesch E, Eberhagen C, Flenkenthaler F, Fröhlich T, Bracher F, Grimm C, et al. Lysosomal TRPML1 regulates mitochondrial function in hepatocellular carcinoma cells. J Cell Sci. 2022;135:. PMID: 35274126. doi:10.1242/jcs.259455. PMC8977057 35274126

[44] Geisslinger F, Müller M, Chao YK, Grimm C, Vollmar AM, Bartel K. Targeting TPC2 sensitizes acute lymphoblastic leukemia cells to chemotherapeutics by impairing lysosomal function. Cell Death Dis. 2022;13:668. PMID: 35915060. doi:10.1038/s41419-022-05105-z. 35915060 PMC9343397

[45] Otsu W, Ishida K, Nakamura S, Shimazawa M, Tsusaki H, Hara H. Blue light-emitting diode irradiation promotes transcription factor EB-mediated lysosome biogenesis and lysosomal cell death in murine photoreceptor-derived cells. Biochem Biophys Res Commun. 2020;526:479–484. PMID: 32234235. doi:10.1016/j.bbrc.2020.03.118. 32234235

[46] Gómez-Sintes R, Ledesma MD, Boya P. Lysosomal cell death mechanisms in aging. Ageing Res Rev. 2016;32:150–168. PMID: 26947122. doi:10.1016/j.arr.2016.02.009. 26947122

[47] Xie Z, Zhao M, Yan C, Kong W, Lan F, Zhao S, Yang Q, Bai Z, Qing H, Ni J, Narengaowa . Cathepsin B in programmed cell death machinery: mechanisms of execution and regulatory pathways. Cell Death Dis 2023;14:255. PMID: 37031185. doi:10.1038/s41419-023-05786-0. 37031185 PMC10082344

[48] Ji Y, Li M, Chang M, Liu R, Qiu J, Wang K, Deng C, Shen Y, Zhu J, Wang W, et al. Inflammation: roles in skeletal muscle atrophy. Antioxidants (Basel). 2022;11:1686. PMID: 36139760. doi:10.3390/antiox11091686. 36139760 PMC9495679

[49] McBrayer M, Nixon RA. Lysosome and calcium dysregulation in alzheimer’s disease: partners in crime. Biochem Soc Trans. 2013;41:1495–1502. PMID: 24256243. doi:10.1042/BST20130201. 24256243 PMC3960943

[50] Abuarab N, Munsey TS, Jiang LH, Li J, Sivaprasadarao A. High glucose-induced ros activates TRPM2 to trigger lysosomal membrane permeabilization and Zn(2+)-mediated mitochondrial fission. Sci Signal. 2017;10:eaal4161. PMID: 28765513. doi:10.1126/scisignal.aal4161. 28765513

[51] Rojas-Rivera D, Hetz C. Tmbim protein family: ancestral regulators of cell death. Oncogene. 2015;34:269–280. PMID: 24561528. doi:10.1038/onc.2014.6. 24561528

[52] Pihán P, Lisbona F, Borgonovo J, Edwards-Jorquera S, Nunes-Hasler P, Castillo K, Kepp O, Urra H, Saarnio S, Vihinen H, et al. Control of lysosomal-mediated cell death by the pH-dependent calcium channel RECS1. Sci Adv. 2021;7:eabe5469. PMID: 34767445. doi:10.1126/sciadv.abe5469. 34767445 PMC8589314

[53] Hannun YA, Obeid LM. Sphingolipids and their metabolism in physiology and disease. Nat Rev Mol Cell Biol. 2018;19:175–191. PMID: 29165427. doi:10.1038/nrm.2017.107. 29165427 PMC5902181

[54] Abed Rabbo M, Khodour Y, Kaguni LS, Stiban J. Sphingolipid lysosomal storage diseases: from bench to bedside. Lipids Health Dis. 2021;20:44. PMID: 33941173. doi:10.1186/s12944-021-01466-0. 33941173 PMC8094529

[55] Boer DEC, van Smeden J, Bouwstra JA, Aerts J. Glucocerebrosidase: functions in and beyond the lysosome. J Clin Med. 2020;9:736. PMID: 32182893. doi:10.3390/jcm9030736. 32182893 PMC7141376

[56] Breiden B, Sandhoff K. Acid sphingomyelinase, a lysosomal and secretory phospholipase C, is key for cellular phospholipid catabolism. Int J Mol Sci. 2021;22:9001. PMID: doi:10.3390/ijms22169001. 34445706 PMC8396676

[57] Ehlert K, Frosch M, Fehse N, Zander A, Roth J, Vormoor J. Farber disease: clinical presentation, pathogenesis and a new approach to treatment. Pediatr Rheumatol Online J. 2007;5:15. PMID: 17603888. doi:10.1186/1546-0096-5-15. 17603888 PMC1920510

[58] López-Montero I, Monroy F, Vélez M, Devaux PF. Ceramide: from lateral segregation to mechanical stress. Biochim Biophys Acta. 2010;1798:1348–1356. PMID: 20026045. doi:10.1016/j.bbamem.2009.12.007. 20026045

[59] Pinto SN, Silva LC, Futerman AH, Prieto M. Effect of ceramide structure on membrane biophysical properties: the role of acyl chain length and unsaturation. Biochim Biophys Acta. 2011;1808:2753–2760. PMID: 21835161. doi:10.1016/j.bbamem.2011.07.023. 21835161

[60] Yasuda T, Al Sazzad MA, Jäntti NZ, Pentikäinen OT, Slotte JP. The influence of hydrogen bonding on sphingomyelin/colipid interactions in bilayer membranes. Biophys J. 2016;110:431–440. PMID: 26789766. doi:10.1016/j.bpj.2015.11.3515. 26789766 PMC4724628

[61] Vaccaro AM, Motta M, Tatti M, Scarpa S, Masuelli L, Bhat M, Vanier MT, Tylki-Szymanska A, Salvioli R. Saposin C mutations in gaucher disease patients resulting in lysosomal lipid accumulation, saposin c deficiency, but normal prosaposin processing and sorting. Hum Mol Genet. 2010;19:2987–2997. PMID: 20484222. doi:10.1093/hmg/ddq204. 20484222

[62] Buccinnà B, Piccinini M, Prinetti A, Scandroglio F, Prioni S, Valsecchi M, Votta B, Grifoni S, Lupino E, Ramondetti C, et al. Alterations of myelin-specific proteins and sphingolipids characterize the brains of acid sphingomyelinase-deficient mice, an animal model of Niemann-Pick disease type A. J Neurochem. 2009;109:105–115. PMID: 19187445. doi:10.1111/j.1471-4159.2009.05947.x. 19187445

[63] Villamil Giraldo AM, Appelqvist H, Ederth T, Öllinger K. Lysosomotropic agents: impact on lysosomal membrane permeabilization and cell death. Biochem Soc Trans. 2014;42:1460–1464. PMID: 25233432. doi:10.1042/bst20140145. 25233432

[64] Platt FM, Boland B, van der Spoel AC. Lysosomal storage disorders: the cellular impact of lysosomal dysfunction. J Cell Biol. 2012;199:723–734. doi:10.1083/jcb.201208152. 23185029 PMC3514785

[65] Finnigan GC, Ryan M, Stevens TH. A genome-wide enhancer screen implicates sphingolipid composition in vacuolar atpase function in *Saccharomyces cerevisiae*. Genetics. 2011;187:771–783. doi:10.1534/genetics.110.125567. 21196517 PMC3063671

[66] van der Poel S, Wolthoorn J, van den Heuvel D, Egmond M, Groux-Degroote S, Neumann S, Gerritsen H, van Meer G, Sprong H. Hyperacidification of trans-Golgi network and endo/lysosomes in melanocytes by glucosylceramide-dependent V-ATPase activity. Traffic. 2011;12:1634–1647. PMID: 21810155. doi:10.1111/j.1600-0854.2011.01263.x. 21810155

[67] Collins MP, Forgac M. Regulation and function of V-ATPases in physiology and disease. Biochim Biophys Acta Biomembr. 2020;1862:183341. PMID: 32422136. doi:10.1016/j.bbamem.2020.183341. 32422136 PMC7508768

[68] Lloyd-Evans E, Platt FM. Lysosomal Ca(2+) homeostasis: role in pathogenesis of lysosomal storage diseases. Cell Calcium. 2011;50:200–205. PMID: 21724254. doi:10.1016/j.ceca.2011.03.010. 21724254

[69] Galione A, Morgan AJ, Arredouani A, Davis LC, Rietdorf K, Ruas M, Parrington J. NAADP as an intracellular messenger regulating lysosomal calcium-release channels. Biochem Soc Trans. 2010;38:1424–1431. PMID: 21118101. doi:10.1042/bst0381424. 21118101

[70] Waller-Evans H, Lloyd-Evans E. Regulation of trpml1 function. Biochem Soc Trans. 2015;43:442–446. PMID: 26009188. doi:10.1042/bst20140311. 26009188

[71] Darios F, Stevanin G. Impairment of lysosome function and autophagy in rare neurodegenerative diseases. J Mol Biol. 2020;432:2714–2734. PMID: 32145221. doi:10.1016/j.jmb.2020.02.033. 32145221 PMC7232018

[72] Ogretmen B. Sphingolipid metabolism in cancer signalling and therapy. Nat Rev Cancer. 2018;18:33–50. PMID: 29147025. doi:10.1038/nrc.2017.96. 29147025 PMC5818153

[73] de Duve C, de Barsy T, Poole B, Trouet A, Tulkens P, Van Hoof F. Commentary. Lysosomotropic agents. Biochem Pharmacol. 1974;23:2495–2531. doi:10.1016/0006-2952(74)90174-9. PMID: 4606365. 4606365

[74] Pisonero-Vaquero S, Medina DL. Lysosomotropic drugs: pharmacological tools to study lysosomal function. Curr Drug Metab. 2017;18:1147–1158. PMID: 28952432. doi:10.2174/1389200218666170925125940. 28952432

[75] Thiele DL, Lipsky PE. Mechanism of L-leucyl-L-leucine methyl ester-mediated killing of cytotoxic lymphocytes: dependence on a lysosomal thiol protease, dipeptidyl peptidase I, that is enriched in these cells. Proc Natl Acad Sci U S A. 1990;87:83–87. PMID: 2296607. doi:10.1073/pnas.87.1.83. 2296607 PMC53204

[76] Firestone RA, Pisano JM, Bonney RJ. Lysosomotropic agents. 1. Synthesis and cytotoxic action of lysosomotropic detergents. J Med Chem. 1979;22:1130–1133. PMID: 114658. doi:10.1021/jm00195a026. 114658

[77] Dubowchik GM, Gawlak SL, Firestone RA. The *in vitro* effects of three lysosomotropic detergents against three human tumor cell lines. Bioorg Med Chem Lett. 1995;5:893–898. doi:10.1016/0960-894X(95)00136-H.

[78] Kornhuber J, Tripal P, Reichel M, Mühle C, Rhein C, Muehlbacher M, Groemer TW, Gulbins E. Functional inhibitors of acid sphingomyelinase (FIASMAS): a novel pharmacological group of drugs with broad clinical applications. Cell Physiol Biochem. 2010;26:9–20. PMID: 20502000. doi:10.1159/000315101. 20502000

[79] Boya P, Andreau K, Poncet D, Zamzami N, Perfettini JL, Metivier D, Ojcius DM, Jäättelä M, Kroemer G. Lysosomal membrane permeabilization induces cell death in a mitochondrion-dependent fashion. J Exp Med. 2003;197:1323–1334. PMID: 12756268. doi:10.1084/jem.20021952. 12756268 PMC2193790

[80] Gilbert DN. Aminoglycosides. In: Mandell GL, Bennett JE, Dolin R, eds. Principles and practice of infectious diseases. 4th ed. New York: Churchill Livingstone, 1995. p. 279–301.

[81] Denamur S, Tyteca D, Marchand-Brynaert J, Van Bambeke F, Tulkens PM, Courtoy PJ, Mingeot-Leclercq MP. Role of oxidative stress in lysosomal membrane permeabilization and apoptosis induced by gentamicin, an aminoglycoside antibiotic. Free Radic Biol Med. 2011;51:1656–1665. PMID: 21835240. doi:10.1016/j.freeradbiomed.2011.07.015. 21835240

[82] Nadanaciva S, Lu S, Gebhard DF, Jessen BA, Pennie WD, Will Y. A high content screening assay for identifying lysosomotropic compounds. Toxicol In Vitro. 2011;25:715–723. doi:10.1016/j.tiv.2010.12.010. 21184822

[83] Matsuda S, Okada N, Kodama T, Honda T, Iida T. A cytotoxic type III secretion effector of vibrio parahaemolyticus targets vacuolar H+-ATPase subunit c and ruptures host cell lysosomes. PLoS Pathog. 2012;8:e1002803. PMID: 22829766. doi:10.1371/journal.ppat.1002803. 22829766 PMC3400558

[84] Bewley MA, Naughton M, Preston J, Mitchell A, Holmes A, Marriott HM, Read RC, Mitchell TJ, Whyte MK, Dockrell DH. Pneumolysin activates macrophage lysosomal membrane permeabilization and executes apoptosis by distinct mechanisms without membrane pore formation. mBio. 2014;5:e01710-14. PMID: 25293758. doi:10.1128/mBio.01710-14. 25293758 PMC4196231

[85] Malet JK, Cossart P, Ribet D. Alteration of epithelial cell lysosomal integrity induced by bacterial cholesterol-dependent cytolysins. Cell Microbiol. 2017;19:e12682. PMID: 27739224. doi:10.1111/cmi.12682. 27739224 PMC5347955

[86] Shaughnessy LM, Hoppe AD, Christensen KA, Swanson JA. Membrane perforations inhibit lysosome fusion by altering pH and calcium in listeria monocytogenes vacuoles. Cell Microbiol. 2006;8:781–792. PMID: 16611227. doi:10.1111/j.1462-5822.2005.00665.x. 16611227 PMC1435990

[87] Nabatov AA, Hatzis P, Rouschop KM, van Diest P, Vooijs M. Hypoxia inducible NOD2 interacts with 3-O-sulfogalactoceramide and regulates vesicular homeostasis. Biochim Biophys Acta. 2013;1830:5277–5286. PMID: 23880069. doi:10.1016/j.bbagen.2013.07.017. 23880069

[88] Daussy CF, Wodrich H. “Repair me if you can”: membrane damage, response, and control from the viral perspective. Cells. 2020;9:2042. PMID: 32906744. doi:10.3390/cells9092042. 32906744 PMC7564661

[89] Ludwig IS, Lekkerkerker AN, Depla E, Bosman F, Musters RJ, Depraetere S, van Kooyk Y, Geijtenbeek TB. Hepatitis C virus targets DC-SIGN and L-SIGN to escape lysosomal degradation. J Virol. 2004;78:8322–8332. PMID: 15254204. doi:10.1128/jvi.78.15.8322-8332.2004. 15254204 PMC446128

[90] Ju X, Yan Y, Liu Q, Li N, Sheng M, Zhang L, Li X, Liang Z, Huang F, Liu K, et al. Neuraminidase of influenza a virus binds lysosome-associated membrane proteins directly and induces lysosome rupture. J Virol. 2015;89:10347–10358. PMID: 26246576. doi:10.1128/jvi.01411-15. 26246576 PMC4580162

[91] Peng X, Dela Cruz CS, Sharma L. Coronaviruses, lysosomes, and secondary bacterial infections: coronaviruses outsmart the host. DNA Cell Biol. 2023;42:189–193. PMID: 36763591. doi:10.1089/dna.2023.0002. 36763591

[92] Wang W-A, Carreras-Sureda A, Demaurex N. SARS-CoV-2 infection alkalinizes the ergic and lysosomes through the viroporin activity of the viral envelope protein. J Cell Sci. 2023;136:jcs260685. doi:10.1242/jcs.260685. 36807531 PMC10112968

[93] Levy JMM, Towers CG, Thorburn A. Targeting autophagy in cancer. Nat Rev Cancer. 2017;17:528–542. doi:10.1038/nrc.2017.53. 28751651 PMC5975367

[94] Trybus W, Trybus E, Król T. Lysosomes as a target of anticancer therapy. Int J Mol Sci. 2023;24:2176. doi:10.3390/ijms24032176. 36768500 PMC9916765

[95] Marino J, García Vior MC, Furmento VA, Blank VC, Awruch J, Roguin LP. Lysosomal and mitochondrial permeabilization mediates zinc(II) cationic phthalocyanine phototoxicity. Int J Biochem Cell Biol. 2013;45:2553–2562. PMID: 23994488. doi:10.1016/j.biocel.2013.08.012. 23994488

[96] Kessel D, Luo Y, Mathieu P, Reiners JJ Jr. Determinants of the apoptotic response to lysosomal photodamage. Photochem Photobiol. 2000;71:196–200. PMID: 10687394. doi:10.1562/0031-8655(2000)071<0196:dotart>2.0.co;2. 10687394

[97] Caruso JA, Mathieu PA, Joiakim A, Leeson B, Kessel D, Sloane BF, Reiners JJ Jr. Differential susceptibilities of murine hepatoma 1C1C7 and Tao cells to the lysosomal photosensitizer NPe6: influence of aryl hydrocarbon receptor on lysosomal fragility and protease contents. Mol Pharmacol. 2004;65:1016–1028. PMID: 15044632. doi:10.1124/mol.65.4.1016. 15044632

[98] Ichinose S, Usuda J, Hirata T, Inoue T, Ohtani K, Maehara S, Kubota M, Imai K, Tsunoda Y, Kuroiwa Y, et al. Lysosomal cathepsin initiates apoptosis, which is regulated by photodamage to BCL-2 at mitochondria in photodynamic therapy using a novel photosensitizer, ATX-s10 (Na). Int J Oncol. 2006;29:349–355. PMID: 16820876. 16820876

[99] Yamashita G, Takano N, Kazama H, Tsukahara K, Miyazawa K. P53 regulates lysosomal membrane permeabilization as well as cytoprotective autophagy in response to DNA-damaging drugs. Cell Death Discov. 2022;8:502. doi:10.1038/s41420-022-01293-x. 36581628 PMC9800408

[100] Santos S, Persechini PM, Henriques-Santos BM, Bello-Santos VG, Castro NG, Costa de Sousa J, Genta FA, Santiago MF, Coutinho-Silva R, Savio LEB, et al. P2x7 receptor triggers lysosomal leakage through calcium mobilization in a mechanism dependent on pannexin-1 hemichannels. Front Immunol. 2022;13:752105. PMID: 35222364. doi:10.3389/fimmu.2022.752105. 35222364 PMC8863609

[101] Halaby R. Natural products induce lysosomal membrane permeabilization as an anticancer strategy. Medicines (Basel). 2021;8:69. PMID: 34822366. doi:10.3390/medicines8110069. 34822366 PMC8624533

[102] Schütz I, Lopez-Hernandez T, Gao Q, Puchkov D, Jabs S, Nordmeyer D, Schmudde M, Rühl E, Graf CM, Haucke V. Lysosomal dysfunction caused by cellular accumulation of silica nanoparticles. J Biol Chem. 2016;291:14170–14184. PMID: 27226546. doi:10.1074/jbc.M115.710947. 27226546 PMC4933175

[103] Vietri M, Radulovic M, Stenmark H. The many functions of escrts. Nat Rev Mol Cell Biol. 2020;21:25–42. doi:10.1038/s41580-019-0177-4. 31705132

[104] Skowyra ML, Schlesinger PH, Naismith TV, Hanson PI. Triggered recruitment of escrt machinery promotes endolysosomal repair. Science. 2018;360:eaar5078. doi:10.1126/science.aar5078. 29622626 PMC6195421

[105] Radulovic M, Schink KO, Wenzel EM, Nähse V, Bongiovanni A, Lafont F, Stenmark H. ESCRT-mediated lysosome repair precedes lysophagy and promotes cell survival. EMBO J. 2018;37:e99753. PMID: 30314966. doi:10.15252/embj.201899753. 30314966 PMC6213280

[106] Jia J, Claude-Taupin A, Gu Y, Choi SW, Peters R, Bissa B, Mudd MH, Allers L, Pallikkuth S, Lidke KA, et al. Galectin-3 coordinates a cellular system for lysosomal repair and removal. Dev Cell. 2020;52:69–87.e8. PMID: 31813797. doi:10.1016/j.devcel.2019.10.025. 31813797 PMC6997950

[107] Jeong E, Willett R, Rissone A, La Spina M, Puertollano R. TMEM55B links autophagy flux, lysosomal repair, and TFE3 activation in response to oxidative stress. Nat Commun. 2024;15:93. PMID: 38168055. doi:10.1038/s41467-023-44316-6. 38168055 PMC10761734

[108] Tan JX, Finkel T. A phosphoinositide signalling pathway mediates rapid lysosomal repair. Nature. 2022;609:815–821. doi:10.1038/s41586-022-05164-4. 36071159 PMC9450835

[109] Liu X, Ridgway ND. Characterization of the sterol and phosphatidylinositol 4-phosphate binding properties of Golgi-associated OSBP-related protein 9 (ORP9). PLoS One. 2014;9:e108368. PMID: 25255026. doi:10.1371/journal.pone.0108368. 25255026 PMC4177916

[110] Zhou Y, Li S, Mäyränpää MI, Zhong W, Bäck N, Yan D, Olkkonen VM. OSBP-related protein 11 (ORP11) dimerizes with ORP9 and localizes at the Golgi-late endosome interface. Exp Cell Res. 2010;316:3304–3316. PMID: 20599956. doi:10.1016/j.yexcr.2010.06.008. 20599956

[111] Antonny B, Bigay J, Mesmin B. The oxysterol-binding protein cycle: Burning off PI(4)P to transport cholesterol. Annu Rev Biochem. 2018;87:809–837. PMID: 29596003. doi:10.1146/annurev-biochem-061516-044924. 29596003

[112] Moser von Filseck J, Čopič A, Delfosse V, Vanni S, Jackson CL, Bourguet W, Drin G. Intracellular transport. Phosphatidylserine transport by orp/osh proteins is driven by phosphatidylinositol 4-phosphate. Science. 2015;349:432–436. PMID: 26206936. doi:10.1126/science.aab1346. 26206936

[113] Mesmin B, Bigay J, Moser von Filseck J, Lacas-Gervais S, Drin G, Antonny B. A four-step cycle driven by PI(4)P hydrolysis directs sterol/PI(4)P exchange by the Er-Golgi tether OSBP. Cell. 2013;155:830–843. PMID: 24209621. doi:10.1016/j.cell.2013.09.056. 24209621

[114] Radulovic M, Wenzel EM, Gilani S, Holland LK, Lystad AH, Phuyal S, Olkkonen VM, Brech A, Jäättelä M, Maeda K, et al. Cholesterol transfer via endoplasmic reticulum contacts mediates lysosome damage repair. EMBO J. 2022;41:e112677. PMID: 36408828. doi:10.15252/embj.2022112677. 36408828 PMC9753466

[115] Osawa T, Ishii Y, Noda NN. Human ATG2B possesses a lipid transfer activity which is accelerated by negatively charged lipids and WIPI4. Genes Cells. 2020;25:65–70. PMID: 31721365. doi:10.1111/gtc.12733. 31721365

[116] Valverde DP, Yu S, Boggavarapu V, Kumar N, Lees JA, Walz T, Reinisch KM, Melia TJ. ATG2 transports lipids to promote autophagosome biogenesis. J Cell Biol. 2019;218:1787–1798. PMID: 30952800. doi:10.1083/jcb.201811139. 30952800 PMC6548141

[117] Osawa T, Kotani T, Kawaoka T, Hirata E, Suzuki K, Nakatogawa H, Ohsumi Y, Noda NN. ATG2 mediates direct lipid transfer between membranes for autophagosome formation. Nat Struct Mol Biol. 2019;26:281–288. PMID: 30911189. doi:10.1038/s41594-019-0203-4. 30911189

[118] Niekamp P, Scharte F, Sokoya T, Vittadello L, Kim Y, Deng Y, Südhoff E, Hilderink A, Imlau M, Clarke CJ, et al. Ca(2+)-activated sphingomyelin scrambling and turnover mediate ESCRT-independent lysosomal repair. Nat Commun. 2022;13:1875. PMID: 35388011. doi:10.1038/s41467-022-29481-4. 35388011 PMC8986845

[119] Yim WW, Yamamoto H, Mizushima N. Annexins A1 and A2 are recruited to larger lysosomal injuries independently of escrts to promote repair. FEBS Lett. 2022;596:991–1003. PMID: 35274304. doi:10.1002/1873-3468.14329. 35274304

[120] Cross J, Durgan J, McEwan DG, Tayler M, Ryan KM, Florey O. Lysosome damage triggers direct ATG8 conjugation and ATG2 engagement via non-canonical autophagy. J Cell Biol. 2023;222. PMID: 37796195. doi:10.1083/jcb.202303078. PMC10561555 37796195

[121] Wang F, Peters R, Jia J, Mudd M, Salemi M, Allers L, Javed R, Duque TLA, Paddar MA, Trosdal ES, et al. ATG5 provides host protection acting as a switch in the atg8ylation cascade between autophagy and secretion. Dev Cell. 2023;58:866–884.e8. doi:10.1016/j.devcel.2023.03.014.PMID:39026874. 37054706 PMC10205698

[122] Chauhan S, Kumar S, Jain A, Ponpuak M, Mudd MH, Kimura T, Choi SW, Peters R, Mandell M, Bruun JA, et al. TRIMS and galectins globally cooperate and TRIM16 and galectin-3 co-direct autophagy in endomembrane damage homeostasis. Dev Cell. 2016;39:13–27. PMID: 27693506. doi:10.1016/j.devcel.2016.08.003. 27693506 PMC5104201

[123] Yoshida Y, Yasuda S, Fujita T, Hamasaki M, Murakami A, Kawawaki J, Iwai K, Saeki Y, Yoshimori T, Matsuda N, et al. Ubiquitination of exposed glycoproteins by SCF(FBXO27) directs damaged lysosomes for autophagy. Proc Natl Acad Sci U S A. 2017;114:8574–8579. PMID: 28743755. doi:10.1073/pnas.1702615114. 28743755 PMC5559013

[124] Papadopoulos C, Kirchner P, Bug M, Grum D, Koerver L, Schulze N, Poehler R, Dressler A, Fengler S, Arhzaouy K, et al. VCP/P97 cooperates With YOD1, UBXD1 and PLAA to drive clearance of ruptured lysosomes by autophagy. EMBO J. 2017;36:135–150. PMID: 27753622. doi:10.15252/embj.201695148. 27753622 PMC5242375

[125] Seike T, Terasawa K, Iwata T, Guan JL, Watabe T, Yokoyama S, Hara-Yokoyama M. Site-specific photo-crosslinking of Hsc70 with the KFERQ pentapeptide motif in a chaperone-mediated autophagy and microautophagy substrate in mammalian cells. Biochem Biophys Res Commun. 2024;736:150515. PMID: 39128268. doi:10.1016/j.bbrc.2024.150515. 39128268

[126] Turcu AL, Versini A, Khene N, Gaillet C, Cañeque T, Müller S, Rodriguez R. DMT1 inhibitors kill cancer stem cells by blocking lysosomal iron translocation. Chemistry. 2020;26:7369–7373. PMID: 32083771. doi:10.1002/chem.202000159. 32083771

[127] Plotegher N, Duchen MR. Mitochondrial dysfunction and neurodegeneration in lysosomal storage disorders. Trends Mol Med. 2017;23:116–134. PMID: 28111024. doi:10.1016/j.molmed.2016.12.003. 28111024

